# Multidimensional Clusters of CD4+ T Cell Dysfunction Are Primarily Associated with the CD4/CD8 Ratio in Chronic HIV Infection

**DOI:** 10.1371/journal.pone.0137635

**Published:** 2015-09-24

**Authors:** Juliet Frederiksen, Marcus Buggert, Kajsa Noyan, Piotr Nowak, Anders Sönnerborg, Ole Lund, Annika C. Karlsson

**Affiliations:** 1 Center for Biological Sequence Analysis, Department of Systems Biology, Technical University of Denmark, Lyngby, Denmark; 2 Division of Clinical Microbiology, Department of Laboratory Medicine, Karolinska Institutet, Stockholm, Sweden; 3 Department of Microbiology, University of Pennsylvania, Philadelphia, Pennsylvania, United States of America; 4 Unit of Infectious Diseases, Department of Medicine Huddinge, Karolinska Institutet, Karolinska University Hospital Huddinge, Stockholm, Sweden; Institute of Infection and Global Health, UNITED KINGDOM

## Abstract

HIV infection provokes a myriad of pathological effects on the immune system where many markers of CD4+ T cell dysfunction have been identified. However, most studies to date have focused on single/double measurements of immune dysfunction, while the identification of pathological CD4+ T cell clusters that is highly associated to a specific biomarker for HIV disease remain less studied. Here, multi-parametric flow cytometry was used to investigate immune activation, exhaustion, and senescence of diverse maturation phenotypes of CD4+ T cells. The traditional method of manual data analysis was compared to a multidimensional clustering tool, FLOw Clustering with K (FLOCK) in two cohorts of 47 untreated HIV-infected individuals and 21 age and sex matched healthy controls. In order to reduce the subjectivity of FLOCK, we developed an “artificial reference”, using 2% of all CD4+ gated T cells from each of the HIV-infected individuals. Principle component analyses demonstrated that using an artificial reference lead to a better separation of the HIV-infected individuals from the healthy controls as compared to using a single HIV-infected subject as a reference or analyzing data manually. Multiple correlation analyses between laboratory parameters and pathological CD4+ clusters revealed that the CD4/CD8 ratio was the preeminent surrogate marker of CD4+ T cells dysfunction using all three methods. Increased frequencies of an early-differentiated CD4+ T cell cluster with high CD38, HLA-DR and PD-1 expression were best correlated (Rho = -0.80, P value = 1.96×10^−11^) with HIV disease progression as measured by the CD4/CD8 ratio. The novel approach described here can be used to identify cell clusters that distinguish healthy from HIV infected subjects and is biologically relevant for HIV disease progression. These results further emphasize that a simple measurement of the CD4/CD8 ratio is a useful biomarker for assessment of combined CD4+ T cell dysfunction in chronic HIV disease.

## Introduction

Human immunodeficiency virus type 1 (HIV) infection is characterized by an initial loss of CCR5+CD4+ T cells at mucosal sites of the body [1, 2], and later a gradual decline of central and effector memory CD4+ T cells due to high cell turnover [3], pyroptosis [4], apoptosis [5–7] and/or many other effects that impair normal immune homeostasis [3, 8–10]. Except from becoming infected with HIV, CD4+ T cells also exhibit numerous pathological changes that are contributors, or consequences, of HIV disease progression. The most classically studied markers of disease progression probably involve CD38 and HLA-DR, which are used as measurements of T cell activation [11]. Immune activation has previously been shown to be highly predictive of HIV disease progression [3, 12] and thought to be directly involved in the process of CD4+ T cell division and depletion [13, 14]. Importantly, CD38 and HLA-DR are elevated in most individuals on long-term combined antiretroviral therapy (ART) and predictive of immune recovery and mortality post ART [15]. PD-1 and Tim-3 are markers of T cell exhaustion where both have been shown to be elevated in dysfunctional T cells after HIV and other chronic viral infections [16–18]. Particularly elevated levels of PD-1, together with CD38 and HLA-DR expression, has previously been demonstrated in European [19] and African [20] cohorts to be highly associated with HIV disease progression, independently of T cell maturation phenotypes. Likewise, the memory phenotypes of CD4+ T cells might be highly skewed, where particularly markers of immunosenescence (CD28- and CD57+ cells) are upregulated in HIV-infected subjects, leading to poor T cell proliferation and homeostasis [21].

As HIV infection primarily affects CD4+ T cells, bulk measurements from heterogeneous samples with *e*.*g*. microarray prevent detailed characterization of immunological sub-populations [22]. Therefore, single-cell analysis tools, such as flow cytometry (FCM), are optimal for cell characterization within HIV research. Advances in the instrumentation and reagents have allowed an increased number of parameters to be measured simultaneously on individual cells, yielding data of high dimensionality and complexity [23–25]. Data analysis of high-dimensional FCM data has long been the caveat of polychromatic flow cytometry experiments [26, 27], where traditional data analysis techniques are not only time-consuming but also highly subjective to the experience of the operator [27–29].

Over the last decade though, there has been an increase in the number of automated data analysis techniques developed for FCM [30, 31]. The approaches can be divided into algorithms for automated subset identification and algorithms that quantify differences between multivariate distributions [32, 33]. The automated subset identification solutions are model-based, graph-based or multidimensional clustering approaches [30, 31]. The model-based methods use applications of Bayes mixture Gaussian models, t-mixture models and skew-t models [30]. These methods show robustness that follows predefined models, but they might be slow at estimating model parameters of high-dimensional dataset. SamSPECTRAL [34] is an example of the graph-based methods, while multidimensional clustering approaches available include SPADE [27], flowMeans [35] and FLOCK [36]. Here, we investigated whether one of the existing multidimensional clustering approaches, FLOCK, could delineate HIV-infected *versus* healthy control subjects based on the measurement of the eight markers of CD4+ T cell memory (CD45RO, CD27), activation (CD38, HLA-DR), exhaustion (PD-1, Tim-3) and senescence (CD28, CD57). FLOCK was chosen for these analyses due to its ability to determine centroids of a reference sample and apply these to other samples, and therefore making the results directly comparable between all samples. After developing an “artificial reference” patient, using gated CD4+ T cell data from the HIV-infected subjects; we investigated if FLOCK was 1) superior in delineating the HIV-infected from the healthy control group compared to normal manual data analysis and 2) which traditional HIV disease biomarker that was associated with the multidimensional clusters of CD4+ T cells dysfunction in chronic HIV disease.

## Materials and Methods

### Ethical statement

The Regional Ethical Review Board (Stockholm, Sweden, Dnr 2009-1485-31-3) approved the study. Written informed consent of all study subjects was documented in accordance with the Declaration of Helsinki and all participants were provided with written and oral information about the study.

### Study subjects

In total, 47 HIV-infected individuals (HIV+) were recruited from the Karolinska University Hospital Huddinge, Stockholm, Sweden. All patient samples were collected from untreated subjects; except for three individuals with AIDS defining illnesses (AIDS patients), which viral load measurements were excluded from the statistical analysis. An age and sex matched healthy control group (n = 21) was recruited to compare the CD4+ T cell clusters (Table 1). This cohort has partly been used in a previous study by Buggert et al [19], where detailed information about the HIV-infected subjects are described [19].

**Table 1:** Summary of the HIV-infected cohort.

Character	HIV-infected cohort
n	47
Age, years	40 (33–49)
Males, n (%)	31 Males (66%)
Females, n (%)	16 Females (33%)
CD4 counts, cells/μl	390 (310–500)
CD4%	23 (17–31)
CD8 counts, cells/μl	900 (610–1211)
CD8%	49 (43–60)
CD4/CD8 ratio	0.44 (0.27–0.77)
Viral load, copies/ml	33800 (1138–990000)

Median (IQR) shown for all parameters; n: numbers.

### Antibody reagents

A single flow cytometry panel was tested on all HIV-infected individuals and healthy control subjects as seen in Buggert et al. The antibodies used in the assay were: anti-CD3 APC-H7 (Clone SK7), anti-CD14 V500 (Clone M5E2), anti-CD19 V500 (Clone B43) (BD Bioscience); anti-CD45RO ECD (clone UCHL1) (Beckman Coulter); anti-CD4 BV650 (Clone OKT4), anti-PD-1 BV421 (clone EH12.2H7), anti-CD27 BV785 (clone O323), anti-CD28 PerCP-Cy5.5 (clone CD28.2), anti-CD38 APC (clone HIT2), anti-CD57 FITC (clone HCD57) (Biolegend); anti-HLA-DR PE-Cy7 (clone LN3) (eBioscience); anti-CD8 Qd565 (Clone 3B5) (Life Technologies) and anti-Tim-3 PE (clone 344823) (R&D Systems). LIVE/DEAD Aqua amine dye (Life Technologies) was used to discriminate dead cells and debris.

### Cells and flow cytometric staining

Peripheral blood mononuclear cells (PBMC) were isolated from EDTA collected whole blood by Hypaque-Ficoll (GE Healthcare) density gradient centrifugation and then cryopreserved in 90% FBS (Life Technologies) supplemented with DMSO (Sigma Aldrich). The cells were thawed, washed and rested overnight at 37°C, in R10 (RPMI-1640 Medium AQmedia (Sigma Aldrich) containing 10% FBS, 50 IU/mL penicillin and 50 μg/mL streptomycin, and 10 mM HEPES (Life Technologies)). The PBMCs were counted the next day using a Nucleocounter (ChemoMetec A/S) and added to a concentration of 1.5 × 10^6^ cells/well in V-bottom plates. Cells were washed in PBS, containing 2mM EDTA, and stained with all antibody reagents for 30 min at 20°C as previously described [37]. Cells were washed and resuspended in PBS containing 1% paraformaldehyde (PFA). All flow cytometric analyses were conducted within 2hrs after fixation.

### Flow cytometric analyses

PBMC were analysed on a LSR Fortessa (BD Biosciences) where minimally 600,000 total events were collected per run. Data compensation was performed using antibody capture beads (BD Biosciences) after separate stainings with all antibodies used in the experiment. FlowJo 8.8.7 (Treestar) were used for flow cytometric gating analyses and all manual gates were based on fluorescence minus one (FMO) staining [38].

### Generation of the artificial reference

The manual gating of the FCM data produced CD4+ T cell gated data. This data was used to create the artificial patient. The median number of CD4+ T cells was calculated from the HIV+ data files. The desirable percentage events that were needed to be collected from each of the HIV+ data files was calculated, $\frac{\text{median}\left(\#\text{CD}4+\text{ events}\right)}{\text{total}\left(\#\text{CD}4+\text{ events}\right)}*100$ Thereby, a random 2% of the files were collected to create an artificial HIV+ subject. The artificial HIV+ subject was used to create the centroid table and this was applied to the entire dataset, including both the HIV+ and control data files.

### Data analysis

Heat maps in conjunction with unsupervised hierarchical clustering were used as a method for data visualization. The distance measure used for the hierarchical clustering is the dissimilarity index *dist*_i,j_ = 1-cor(p_i_,p_j_), where *cor* is the Spearman rank correlation calculated for p_i_ and p_j_ is the population frequency vector for subject *i* and *j*, respectively. The Kolmogorov-Smirnov (KS) test statistic is used to quantify the differences between two sample distributions to determine whether they were drawn from the same distribution. Experimental variables between healthy controls and HIV-infected individuals were analyzed using Mann-Whitney U test. Correlations were assessed using non-parametric Spearman rank tests. Bonferroni corrections were applied to all cases where multiple testing was performed. To summarize, flow cytometry data was analysed with FlowJo 8.8.7 (Treestar), FLOCK (ImmPort, NIH web site www.immport.org) and R environment [39].

## Results

**Fig 1:**
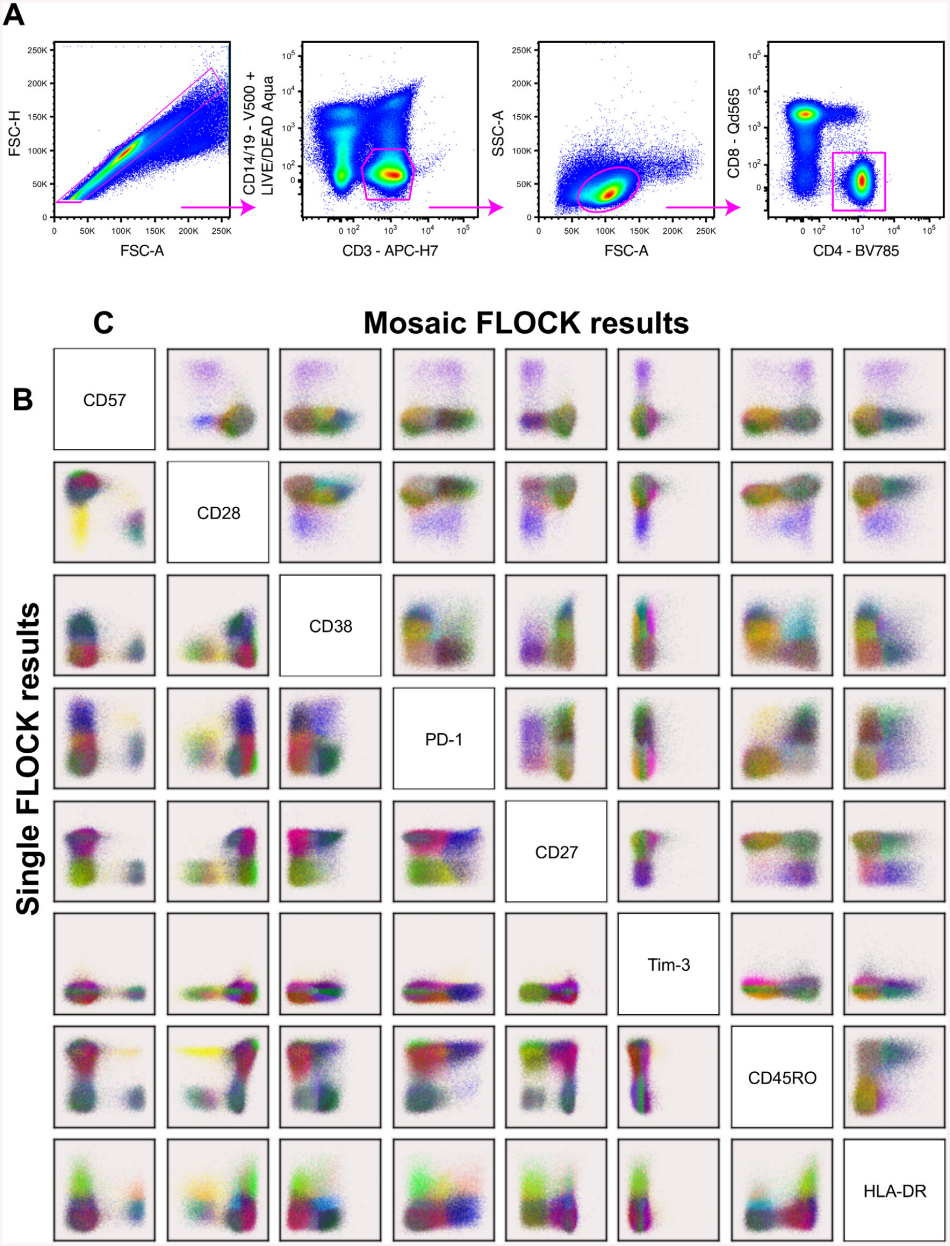
Flow cytometry gating and FLOCK populations. The manual gating strategy used to gate for the CD4+ T cells is shown in the top panel (A). The CD4+ T cell events were uploaded to immPort (immport.niaid.nih.gov) for FLOCK analysis. The unique populations identified by FLOCK using the single HIV reference (bottom left) (B) and artificial reference (top right) (C) is shown. The artificial reference is made-up of 2% of the CD4+ T cells from each subject in the HIV cohort, where as the single HIV reference is made-up of the CD4+ T cells from a single individual from the HIV cohort that appeared to be biologically representative of the cohort.

FLOCK was used for automated population (cluster) identification to analyze an eight-parameter dataset of HIV+ and healthy control subjects. The eight measured parameters were selected to give an overview of the activation (CD38, HLA-DR), exhaustion (PD-1, Tim-3), senescence (CD28, CD57) and memory differentiation (CD45RO, CD27) status of CD4+ T cells. A traditional gating strategy for isolating CD4+ T cells were performed on the multiparametric flow cytometry data set prior to FLOCK data examination (Fig 1A). Thereafter, FLOCK analyses were performed on all of the HIV-infected subjects, where the number of clusters automatically identified by FLOCK differed between the subjects, ranging from 12–23 clusters. In the interest of being able to compare the data, a reference subject that identified the biologically relevant cell clusters was chosen and the centroids of these clusters were then applied to the remaining healthy controls and HIV-infected subjects. The unique clusters identified in the eight-dimensional space for the representative subject is shown in Fig 1B.

### The development of an artificial reference to reduce the subjectivity

The need for a reference subject in the multidimensional clustering analyses is a source of bias in the FLOCK algorithm, as there is no automated method of selecting the optimal reference subject. Instead as a first step using FLOCK, a single subject is selected to function as a reference in the FLOCK analysis. To remove this subjectivity, a “mosaic” subject was created as an artificial control from a subset of HIV-infected subjects in the study. A random 2% of events from each of the 47 HIV-infected individuals were extracted and concatenated to create the artificial patient. This was hypothesized to give a representative view of the relative phenotypes found in the HIV-infected subjects. The following abbreviations will be used for the remainder of the article; the FLOCK analysis using the specific reference subject that identified the biologically relevant cell clusters will be referred to as sFLOCK and the FLOCK analysis using the artificial reference will be referred to as aFLOCK.

The distinctive clusters identified by sFLOCK (Fig 1B) yielded biologically accountable clusters, similar to those observed in aFLOCK (Fig 1C). The number of bins automatically determined by FLOCK for aFLOCK was greater than that found for sFLOCK, which leads to a larger number of aFLOCK clusters (n = 25) compared to sFLOCK (n = 21) as well as the largest number of clusters determined by any individual (n = 23). This agrees with our expectations, as the method would be sampling from a larger repertoire of cells. The clusters obtained from sFLOCK were shown to be tighter, where the synonymous clusters were more diffuse, which is most likely due to the events being an artificial drawn from different samples. Additionally, the aFLOCK clusters were shown to make biological sense and not artifacts from combining the different samples.

### aFLOCK provides a better separation of HIV-infected subjects and healthy controls

In order to assess the performance of aFLOCK, it was compared to the results of the sFLOCK and manual data analysis. The format of the manual data analysis results is a frequency table with rows of the subjects used in the study and columns for the cell populations investigated (*e*.*g*. CD4+CD38+HLA-DR+, CD4+CD57+CD28-, etc). The results of the FLOCK analyses are tables of cell population frequencies for all study subjects.

**Fig 2:**
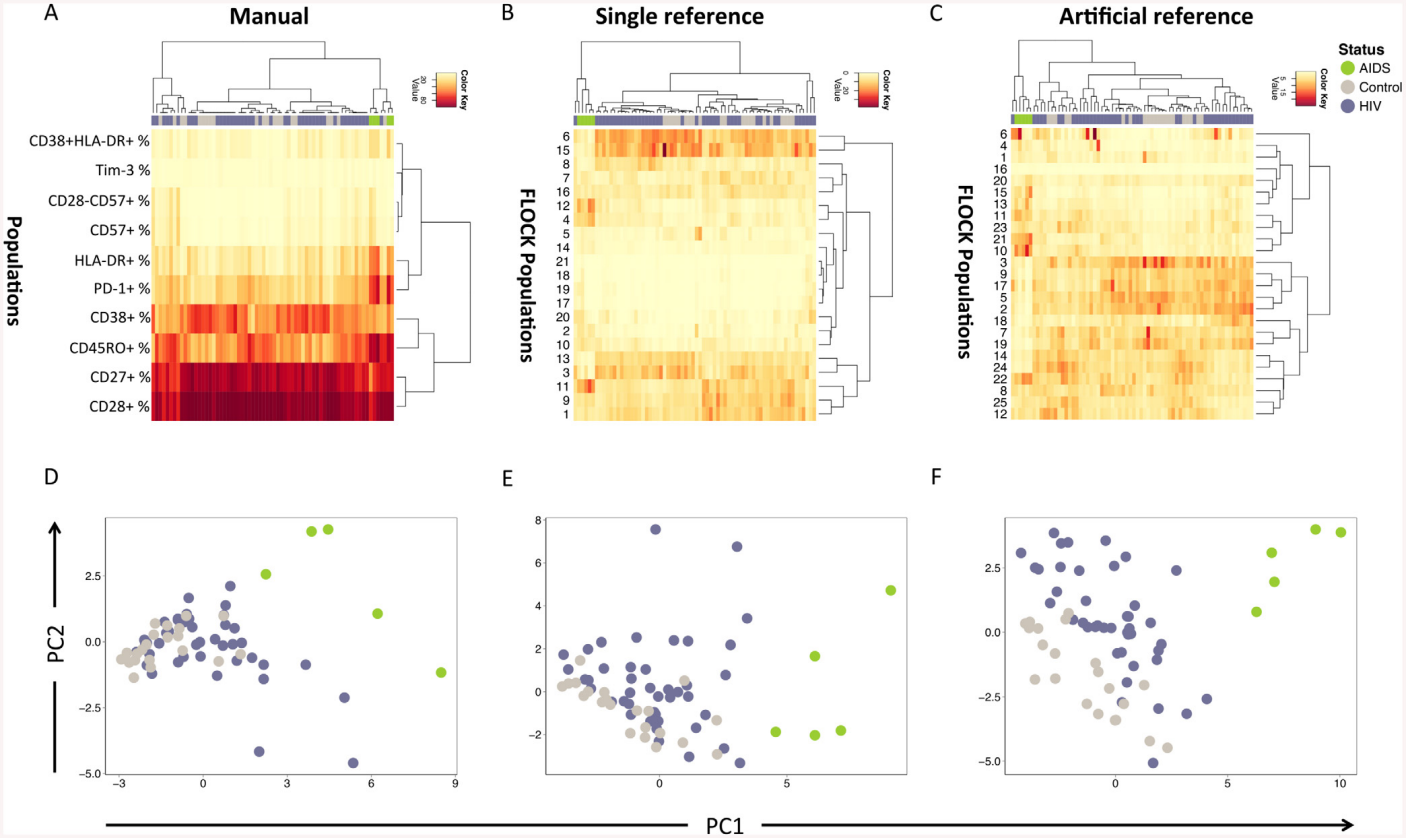
Clustering of HIV-infected and -uninfected subjects with manual and FLOCK gating principles. The top panel shows the heat map representation of the matrices containing the cell population frequencies of the manual gating results (A), the FLOCK results using one HIV infected subject that identified biologically relevant cell populations (B) and the FLOCK results using an artificial of the HIV-infected subjects as a reference (C). The bottom panel shows the principle component analysis (PCA) was performed on the matrices illustrated in A-C to investigate whether there were difference between the control, HIV-infected and AIDS subjects. The results of the PCA performed on the manually determined population frequencies is shown in (D), the results of the K-S test that compared the HIV infected individuals to the healthy controls for PC1 (P value = 0.0009, D value = 0.495) and PC2 (P value = 0.3, D value = 0.236) are shown below the biplot. The FLOCK data using one HIV infected subject that identified biologically relevant cell populations is shown in (E), the results of the K-S test that compared the HIV infected individuals to the healthy controls for PC1 (P value = 0.04, D = 0.353) and PC2 (P value = 0.02, D value = 0.378) are shown below the biplot. The FLOCK data using an artificial of the HIV-infected subjects as a reference is shown in (F), the results of the K-S test that compared the HIV infected individuals to the healthy controls for PC1 (P value = 0.02, D value = 0.384) and PC2 (P value = 0.0008, D value = 0.497) are shown below the biplot. A detailed overview of the FLOCK populations in (B) and (C) can be seen in S1 Table.

Unsupervised hierarchical clustering in conjunction with heat maps, a method for the visualization of numeric matrices to find patterns in data in an unbiased fashion, was used to analyze the three data matrices containing the cluster frequency for row (individual) *i* and column (cluster) *j*. A visualization of the results of the manual and FLOCK FCM data analysis can be seen in Fig 2A–2C. As can visually be noted, the hierarchical clustering of the manual data analysis results failed to separate the HIV-infected individuals and the healthy controls (Fig 2A) as good as sFLOCK (Fig 2B) and aFLOCK (Fig 2C). Notably, the AIDS patients, and an additional HIV-infected subject, were distinguished as outliers in all three methods. It was the same HIV-infected subject, with a low CD4 count and CD4/CD8 ratio comparable to some of the AIDS patients that clustered with the AIDS patients in the FLOCK analyses.

The majority of healthy controls in the aFLOCK results (Fig 2C) were found to cluster in two groups, whereas the sFLOCK (Fig 2B) and manual data analysis (Fig 2A) results showed the clustering of healthy controls to be more spread in comparison. The HIV-infected individual found to cluster with the AIDS patients in the manual data analysis (Fig 2A) had a relatively high CD4/CD8 ratio. In addition to these findings, a healthy control was surprisingly found within this AIDS cluster for the manual data analysis. Overall, these results thus suggest that the aFLOCK captures an immunopathological signal that can more easily separate the HIV-infected individuals from the healthy controls.

To objectively compare the results from the three different FCM data analysis methods, principle component analysis (PCA) of the frequency tables were performed (Fig 2D–2F). It was clearly observed that the AIDS patients were outliers in the PCA biplots of both FLOCK data analyses methods (Fig 2E and 2F), but particularly remarkable in the aFLOCK analysis (Fig 2F). Kolmogorov-Smirnov (KS) tests were used to quantify the distance between two groups to determine whether they were drawn from the same distribution (P-value of 1 for this case). The KS test showed that the distribution of PC1 scores between HIV-infected individuals and control subjects (aFLOCK analysis, Fig 2F) were significantly different (P value = 0.02, D value = 0.384). Similarly for PC2 and the results of the KS test revealed that the HIV+ and control subjects’ distributions were highly significantly different (P value = 0.0008, D value = 0.497). More specifically, it was clusters 5, 10, 2, 11 and 21 (S1 Table) that had the greatest impact on the PC1 scores. These clusters were split between naïve T cell clusters (clusters 2 and 5) and exhausted (± activated) memory T cell clusters (clusters 10, 11 and 21). The PC2 dimension was more efficient at separating the HIV-infected and control subjects, where the clusters that had the largest impact on PC2 were 24, 14, 25, 20 and 12 (mostly naïve T cell clusters). The top 5 clusters that explain PC2 were seen to adhere together in the right part of the heat map, whereas those for PC1 were seen to cluster together in the middle. The KS test for the PCA results of sFLOCK (Fig 2E) showed a significant difference (P value = 0.04, D = 0.353) for the PC1 scores when comparing the distributions of the HIV+ subjects and the healthy controls. A significant difference (P value = 0.02, D value = 0.378) was also seen for the KS test of PC2. In the case of the PCA of the manual FCM data analysis, the KS-test of the PC1 scores for the two groups showed a significant difference (P value = 0.0009, D value = 0.495), however, the PC2 distributions for the two groups did not show a significant difference (P value = 0.3, D value = 0.236).

### The CD4/CD8 ratio is primarily associated with the immunopathogenic clusters

**Fig 3:**
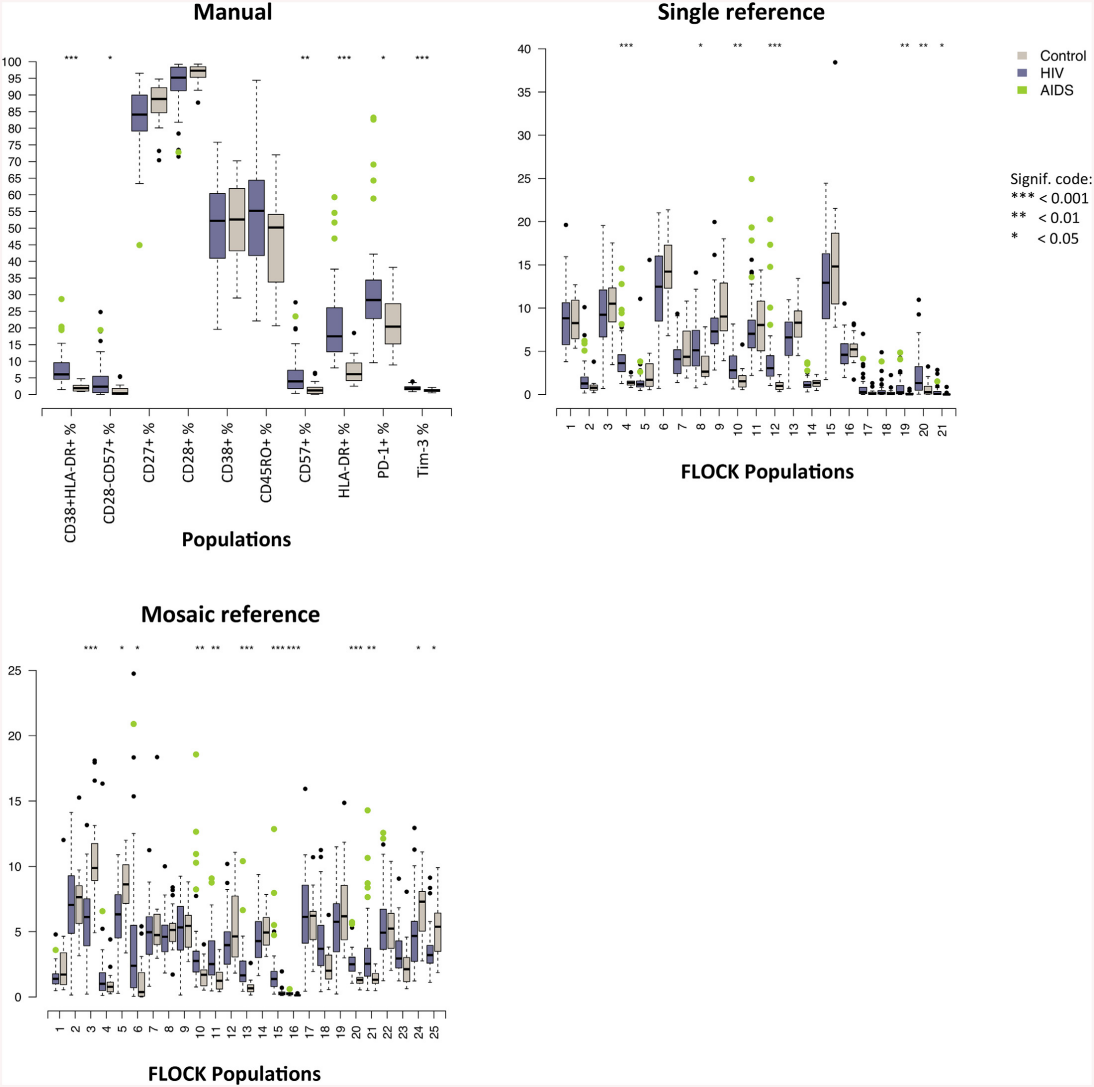
Box plots of all manual and FLOCK gated populations between HIV-infected and -uninfected subjects. Box plot representation of summary results of the two groups generated by the three methods used to investigate the same HIV immunopathogenesis dataset, manual data analysis (left), FLOCK data analysis using the single HIV reference (middle) and FLOCK data analysis using the artificial reference (right). The data is presented in a box and whisker plot where the horizontal line in the box is the median population occupation, the edges of the boxes are the 25th and 75th percentiles of the population occupation and the ‘whiskers’ represent the 10th and 90th percentiles of the population occupation, and the dots indicate outliers. The purple and grey boxes represent the HIV+ and healthy control group, respectively, where the green dots indicate outliers that are AIDS patients. Results of the multiple Mann-Whitney tests followed by Bonferroni adjustments between the HIV and healthy control group for each gated population is shown using P value significance codes found directly above: 0 *** 0.001 ** 0.01 * 0.05.

The FLOCK and manually gated clusters were next correlated to numerous routine laboratory parameters that are used to assess the immune status of HIV-infected individuals. The initial step of the analysis involved the selection for the immunopathogenic clusters for each data set. This was done by performing multiple Mann-Whitney U tests to select for clusters that varied significantly, after Bonferroni correction for multiple testing. The pre-processing step resulted in six clusters (ten clusters before Bonferroni adjustments) for the manual dataset, seven clusters (21 clusters before Bonferroni adjustments) for the sFLOCK dataset and twelve clusters (25 clusters before Bonferroni adjustments) for the aFLOCK dataset. The phenotypes of the sFLOCK and aFLOCK results are shown in S1 Table. Notably, almost half of the original artificial reference dataset captured an immunopathogenic difference between the HIV+ and healthy control groups, whereas only a third of the sFLOCK dataset captured this immunopathogenesis. Box plot representations of these multiple Mann-Whitney U tests are illustrated in Fig 3.

Multiple non-parametric Spearman correlation tests between the immunopathogenic clusters and the routine laboratory parameters were next employed (Table 2). The detailed overview of these results is observed in S2 and S3 Tables. It was observable that the rank of the average absolute Rho-values for the different methods corresponds very well to each other. The average absolute Rho-values were calculated using the union of the immunopathological clusters significantly correlated to any of the clinical markers. In each of the three methods, the CD4/CD8 ratio were shown to have the greatest correlation to the manual or FLOCK gated clusters (Table 2). A close second in each dataset was the CD4%. The CD4% and CD4/CD8 ratio had a correlation coefficient of 0.98 (P value = 2.2×10^−16^) due to the fact that the formula to calculate them differs by an additional denominator, explaining the reason why the average spearman correlation coefficients were very similar.

**Table 2:** Non-parametric Spearman rank tests correlation analysis of the manual, sFLOCK and aFLOCK immunopathological populations to the clinical parameters.

	aFLOCK		sFLOCK		Manual	
	#	R (±SD)^a^	#	R (±SD) ^a^	#	R (±SD) ^a^
CD4 level	4	0.579 (0.131)	2	0.597 (0.191)	3	0.545 (0.037)
CD4%	5	0.622 (0.099)	2	0.633 (0.17)	3	0.586 (0.043)
CD8 level	1	0.384 (0.104)	1	0.385 (0.168)	2	0.404 (0.122)
CD8%	4	0.531 (0.09)	2	0.518 (0.149)	2	0.544 (0.119)
VL	1	0.276 (0.178)	0	0.296 (0.152)	1	0.304 (0.211)
CD4/CD8 ratio	5	0.634 (0.093)	3	0.64 (0.136)	3	0.617 (0.088)

^**a**^ Bonferroni corrections have been performed.

The T cell clusters 15, 21, 10, 11 and 13 significantly correlated with at least one of the laboratory parameters in the aFLOCK dataset. These clusters contained cells that were CD28+, CD38+, PD-1+, CD27+ and CD45RO+. At least one AIDS subject was an extreme outlier in all of these artificial generated clusters and these clusters were shown to be significantly more densely populated for HIV-infected subjects compared to the healthy controls (Fig 3). The majority of the clusters, identified by artificial FLOCK that correlated with the clinical parameters, were memory T cells with multi-dysfunctional traits. Cluster 15 (CD57loCD28hiCD38+PD-1+CD27+Tim-3loCD45ROhiHLA-DR+) was shown to best correlate with all clinical parameters in this data set, where it achieved a correlation of Rho = -0.80 (P value = 1.41×10^−9^) with the CD4/CD8 ratio.

The T cell clusters 4, 10 and 12 significantly correlated with at least one of the clinical parameters for the sFLOCK dataset. These clusters of cells were CD28hi, PD-1+, CD27+, CD45ROhi and HLA-DR+, where AIDS outliers can be seen in Fig 3 for clusters 4 and 12. The activated/exhausted early-differentiated cluster 12 (CD57loCD28hiCD38loPD-1+CD27+Tim-3-CD45ROhiHLA-DR+) of sFLOCK correlated most significantly with the CD4/CD8 ratio (Rho = -0.78, P value = 5.75×10^−9^). This shows that CD4+ T cells with a similar phenotype correlate with HIV disease progression independently of reference subjects. A noteworthy difference however, was that only two sFLOCK clusters correlated with the CD4% whereas three sFLOCK clusters correlated significantly with the CD4/CD8 ratio.

In summary, the manual analysis results showed that activation (CD4+CD38+HLA-DR+, CD4+HLA-DR+) and exhaustion (CD4+PD-1+) markers were primarily correlated with the laboratory parameters, whereas the results from the FLOCK analyses showed that it was specific activation and exhaustion profiles of early-differentiated memory clusters that were linked to the immunopathogenesis of HIV infection. In agreement to what we previously has observed [19], the CD4/CD8 ratio was shown to be the preeminent surrogate marker of pathological CD4+ T cells clusters identified with FLOCK. This data suggest that multidimensional clustering approaches like FLOCK is able to define and correlate specific CD4+ T cell clusters of relevance with HIV disease progression.

## Discussion

Despite the great advances during the past 20 years in reducing AIDS-related mortality, standard therapies do not fully restore health or normal immune status in HIV-infected individuals. Despite many years on therapy, HIV-infected patients maintain elevated levels of immune activation and inflammation, which is linked to the increased incidence of cardiovascular diseases, bone disorders, cognitive impairment and other age-related diseases despite low viral loads and high CD4 counts [40]. Similarly, despite un-detectable viral load and relatively stable high levels of CD4+ T cells, also elite controllers exhibit increased immune activation that is reversed by ART [41]. Thus, it still remains important to understand the dysfunctional changes of the immune system that persists in treated and un-treated HIV-infection.

Due to the myriad events that occur in HIV-infection, it remains highly important to understand the immunopathological changes at the single-cell level. FCM has long been the state-of-the-art method to study this phenomenon [42]. The expression of markers representing the activation (CD38, HLA-DR), exhaustion (PD-1, Tim-3), senescence (CD28, CD57) and memory differentiation (CD45RO, CD27) status of CD4+ T cells were here measured by FCM. Traditionally, FCM data has been analyzed using 1- or 2-dimensional plots sequentially. In this study, FLOCK [36], an algorithm that uses a density-based approach to identify biologically relevant clusters in the multidimensional space, was used to explore the immunopathogenesis of HIV. FLOCK was chosen for its ability to compare the occupation of populations between the two cohorts, the healthy controls and the HIV-infected individuals. During the FlowCAP challenge, FLOCK was shown to perform well in the completely automated challenge [30]. FLOCK was originally used to study B cell subsets found in human PBMC samples, where seventeen B cell subsets (including novel plasmablast subsets) were elucidated. FLOCK has since been used to study a range of different FCM datasets including an investigation into the response kinetics of T cells live-attenuated S. Typhi vaccine [36, 43–47]. To our knowledge, this is the first study that uses FLOCK to investigate a HIV dataset and provide this alternative approach to select a less biased reference for the algorithm.

A reference sample is a requirement for the FLOCK algorithm, from which the cell populations are determined and their centroids are applied to the remaining cohort samples to allow for cross-sample comparisons. A majority of the studies performed a FLOCK analysis of each of their samples and chose the best as a representative sample to define their centroids. The centroids have usually then been applied to all the subjects for a cross sample comparison [36, 45–47]. Although this method has been shown to be the typical way of analyzing FCM data using FLOCK, it imposes a level of subjectivity to the analysis, as one would have to determine the sample that one “feels” gives the best representation of the data. In this study, this normal approach of selecting the centroids was compared to a more automated method, where approximately 2% of each samples were concatenated to create an artificial reference. This artificial reference was used to select the centroids, after which the centroids were applied to all samples. The results showed that the automated method produced a larger number of clusters and a larger proportion of these clusters were shown to be immunopathological, *i*.*e*. different between the HIV-infected and healthy control subjects. This implies that the artificial reference provided a method of capturing a greater view of the pathogenesis distinguished in HIV-infected individuals. Henn et al. [43] used a similar method to prepare the centroids for cross-sample comparisons as the one presented in this article. They concatenated all of the samples in their study to define the centroids before applying them to all samples to study B cell responses in relation to vaccination to influenza. We chose only to use a proportion of the cells extracted from each sample to obtain a reference patient with approximately as many data points as the real patients. This artificial reference generated 25 independent clusters. When the resulting centroids were applied on all samples, twelve of the clusters were shown to be significantly different (after Bonferroni corrections) between the HIV-infected and healthy control subjects. Six of the immunopathological clusters were shown to correlate significantly with both the clinical parameters CD4% and CD4/CD8 ratio. In summary, the CD4/CD8 ratio was shown to correlate most significantly with the immunopathological clusters determined using the manual data analysis and both FLOCK results.

The results from the artificial reference (aFLOCK) were compared to the case of using a single representative individual as a reference for FLOCK (sFLOCK) as well as to the manual data analysis. Interestingly, when all three methods were compared, aFLOCK appeared to improve the separation between healthy controls and HIV-infected individuals in comparison with sFLOCK or manual data analysis. In all three analyses, the first principle component separated the AIDS patients from the remaining HIV-infected individuals and healthy controls, whereas the second principle component was better at separating the HIV-infected individuals from the healthy controls. However, aFLOCK was shown to surpass at separating the distribution of HIV-infected individuals and healthy controls, when compared to the sFLOCK and manual data analysis.

In a previous study on the same cohort, a multi-parametric bioinformatics approach was utilized to determine the immunopathological CD4+ and CD8+ T cell subsets that correlated significantly with the routine laboratory parameters [19]. One of the methods used was Boolean gating, where an early-differentiated CD4+ T cell population expressing activation and exhaustion markers (CD57-CD28+CD38+PD-1+CD27+Tim-3-CD45RO+HLA-DR+) was best correlated with the CD4/CD8 ratio. Numerous previous observations implicate that the homeostatic failure of CD4+ T cell regeneration in chronic HIV infection, is a consequence of a highly dysfunctional and activated pool of early-differentiated CD4+ T cells [47]. This early-differentiated CD4+ T cell population is synonymous to the cluster that aFLOCK identified, (CD57loCD28hiCD38+PD-1+CD27+Tim-3loCD45ROhiHLA-DR+), that correlated most significantly with all the clinical parameters, particularly with the CD4/CD8 ratio. Cluster 12 (CD57loCD28hiCD38loPD-1+CD27+Tim-3-CD45ROhiHLA-DR+) of sFLOCK correlated most significantly to CD4/CD8 ratio with a correlation coefficient of -0.78. Interestingly, the sFLOCK, aFLOCK and Boolean populations that correlated most significantly with the clinical parameters were synonymous. The key difference is that the FLOCK results dispatch a more detailed phenotype of the populations and are not limited to the binary expression of a marker. This is important as it relays a more biologically correct representation of the immune system and offers a less time-consuming way without subjective gating of the populations.

A possible limitation of the study was the exclusion of healthy subjects from the artificial reference, where it would have been interesting to create the artificial reference based on the combined measurements of HIV-infected individuals and healthy controls together. However, we decided to concentrate on the HIV-relevant phenotypes in this study as these subjects most probably were going to generate more extreme populations than healthy controls. Notably some of the HIV-infected individuals were relatively healthy and therefore some of the less dysfunctional subsets should also be present. It is hard to predict how robust this method of ‘selecting’ a reference patient is when a much larger number of subjects are used to create the reference. However, the same issue of whether to pick a healthy, HIV+ or AIDS subject, as a single reference subject would have to be addressed when using the standard FLOCK analysis. It should be iterated that this method of selecting a reference sample could be generalized to analyses where cross-sample comparisons are to be made. A further limitation is the cross-sectional nature of this study, where multiple time points or estimated time from infection would have been highly desirable to *e*.*g*. determine how the CD4/CD8 ratio could predict disease progression or mortality in this cohort. Like previously discussed [19] and mentioned elsewhere [48], we here aimed to conduct an observational study on a highly ethnical diverse population of HIV-infected subjects to determine which traditional biomarker of HIV disease progression that was primarily associated with the dysfunctional CD4+ T cell clusters. Taken together, the measurement of one single time-point is of clinical interest, independently of assessment over time.

The CD4/CD8 ratio has typically been linked in elderly with a general state of immune dysfunction, where an inverted CD4/CD8 ratio is associated with short-time mortality [49–51]. Also in HIV infection the CD4/CD8 ratio has received renewed interest, particularly as it is usually not normalized despite long-term therapy. Older studies have demonstrated that AIDS development in untreated HIV infection is highly predicted by longitudinal assessment of the CD4/CD8 ratio [48], whereas the CD4/CD8 ratio in treated individuals is related to the risk of the comorbidities [52]. In close linkage to our results, a recent report described that a low CD4/CD8 ratio during ART was associated with persistent immune activation and senescent profiles of T cells [53]. Whether the preserved immune activation profile might be a consequence or cause of a low CD4/CD8 ratio is hard to determine, but all these studies together suggest that the CD4/CD8 ratio could be used as a an adequate biomarker for monitoring the state of immune dysfunction and morbidity/mortality in long-term treated HIV-infected subjects. In the wake of studies showing a clear benefit of ART to reduce mortality and transmission levels, possibly all affected individuals in developed countries will be treated in a near future. However, because of socio-economical aspects and other reasons, this seems to be a more distant prospect for developing countries, and therefore an informative biomarker of CD4+ T cell dysfunction and state of HIV disease could also serve as vital information in untreated subjects for a long time ahead. Other studies have demonstrated that the CD4 count is not a perfect predictor of HIV disease progression in HIV-infected individuals from developing countries [54, 55], further emphasizing the role of the CD4/CD8 ratio as a biomarker of interest also in untreated subjects in countries with the major burden of HIV infections.

In this study we have illustrated that the use of an automated clustering algorithm produced better results than the classical manual in separating the HIV-infected individuals and healthy controls. It is important to rely more on the automated methods, as they are less subjective than the manual gating methods. Supplemental to the FLOCK algorithm, we suggest an alternative method for selecting the reference subject for the cross-sample comparison to remove the subjectivity of selecting a reference subject. To sum up, with the growing number of parameters that can be measured with multiparametric flow cytometry it is important to use the automated methods to perform the data analysis as it has been shown in multiple studies that they are just as good, if not better than the classical data analyses.

## Supporting Information

S1 Table Description of the FLOCK population for the single HIV reference and artificial reference. (PDF)

S2 Table Clinical parameters correlating with the populations. (PDF)

S3 Table An in-depth overview of the multiple correlations analysis. (XLSX)
