# Supplementary material for: Multidimensional Clusters of CD4+ T Cell Dysfunction Are Primarily Associated with the CD4/CD8 Ratio in Chronic HIV Infection
Source: PLoS One. 2015 Sep 24;10(9):e0137635. doi: 10.1371/journal.pone.0137635 (PMC4581870; doi:10.1371/journal.pone.0137635)
Supplement: S1 Table — (PDF) [file pone.0137635.s001.pdf]

S1 Table. Description of the FLOCK population for the single HIV reference and artificial reference.

	Single reference		Artificial reference
1	CD57loCD28hiCD38-PD-1loCD27+Tim-3-CD45ROhiHLA-DR-	1	CD57-CD28+CD38loPD-1-CD27loTim-3-CD45ROloHLA-DR-
2	CD57-CD28loCD38-PD-1loCD27-Tim-3-CD45ROhiHLA-DRlo	2	CD57loCD28+CD38loPD-1-CD27+Tim-3-CD45RO-HLA-DR-
3	CD57loCD28+CD38loPD-1loCD27+Tim-3-CD45ROloHLA-DR-	3	CD57-CD28+CD38loPD-1-CD27+Tim-3-CD45RO-HLA-DR-
4	CD57lo**CD28hi**CD38lo**PD-1+CD27+**Tim-3-**CD45ROhi**HLA-DR-	4	CD57loCD28loCD38-PD-1loCD27-Tim-3-CD45RO+HLA-DRlo
5	CD57-CD28loCD38-PD-1loCD27loTim-3-CD45ROloHLA-DR-	5	CD57loCD28+CD38loPD-1-CD27+Tim-3-CD45ROloHLA-DR-
6	CD57loCD28+CD38loPD-1-CD27+Tim-3-CD45ROloHLA-DR-	6	CD57hiCD28loCD38loPD-1+CD27-Tim-3-CD45RO+HLA-DRlo
7	CD57loCD28hiCD38-PD-1loCD27loTim-3-CD45RO+HLA-DR-	7	CD57loCD28+CD38-PD-1-CD27+Tim-3-CD45ROloHLA-DR-
8	CD57loCD28+CD38loPD-1loCD27+Tim-3-CD45ROloHLA-DRlo	8	CD57loCD28hiCD38loPD-1loCD27+Tim-3-CD45RO+HLA-DR-
9	CD57loCD28hiCD38-PD-1loCD27+Tim-3-CD45RO+HLA-DR-	9	CD57loCD28+CD38+PD-1-CD27+Tim-3loCD45RO-HLA-DR-
10	CD57lo**CD28hi**CD38-PD-1loCD27loTim-3-**CD45ROhiHLA-DR+**	10	CD57lo**CD28+**CD38-**PD-1+CD27+**Tim-3-**CD45RO+**HLA-DRlo
11	CD57loCD28hiCD38-PD-1+CD27+Tim-3-CD45ROhiHLA-DRlo	11	CD57lo**CD28hi**CD38-**PD-1+**CD27loTim-3-**CD45ROhiHLA-DR+**
12	CD57lo**CD28hi**CD38lo**PD-1+CD27+**Tim-3-**CD45ROhiHLA-DR+**	12	CD57loCD28hiCD38-PD-1loCD27loTim-3-CD45RO+HLA-DRlo

13	CD57loCD28+CD38loPD-1loCD27+Tim-3loCD45RO-HLA-DR-	13	CD57lo**CD28hi**CD38-**PD-1+CD27+**Tim-3lo**CD45RO+HLA-DR+**
14	CD57loCD28hiCD38loPD-1+CD27+Tim-3loCD45ROhiHLA-DRlo	14	CD57loCD28hiCD38-PD-1loCD27+Tim-3-CD45RO+HLA-DR-
15	CD57loCD28+CD38loPD-1-CD27+Tim-3-CD45ROloHLA-DR-	15	CD57lo**CD28hiCD38+PD-1+CD27+**Tim-3lo**CD45ROhiHLA-DR+**
16	CD57loCD28hiCD38loPD-1loCD27+Tim-3-CD45RO+HLA-DR-	16	CD57loCD28hiCD38loPD-1loCD27+Tim-3loCD45RO+HLA-DR-
17	CD57hiCD28loCD38-PD-1loCD27-Tim-3-CD45ROloHLA-DRlo	17	CD57loCD28+CD38+PD-1loCD27+Tim-3-CD45ROloHLA-DR-
18	CD57hiCD28loCD38-PD-1loCD27-Tim-3-CD45ROloHLA-DR-	18	CD57loCD28+CD38loPD-1-CD27+Tim-3-CD45ROloHLA-DRlo
19	CD57hiCD28loCD38-PD-1loCD27-Tim-3-CD45ROloHLA-DRlo	19	CD57loCD28+CD38loPD-1-CD27+Tim-3loCD45RO-HLA-DR-
20	CD57+CD28+CD38-PD-1+CD27loTim-3-CD45ROhiHLA-DRlo	20	CD57loCD28+CD38loPD-1+CD27+Tim-3-CD45ROloHLA-DR-
21	CD57hiCD28loCD38-PD-1loCD27-Tim-3-CD45RO-HLA-DRlo	21	CD57lo**CD28hiCD38+PD-1+CD27+**Tim-3-**CD45ROhi**HLA-DRlo
		22	CD57loCD28hiCD38-PD-1+CD27+Tim-3-CD45RO+HLA-DR-
		23	CD57loCD28hiCD38-PD-1loCD27+Tim-3-CD45RO+HLA-DRlo
		24	CD57loCD28hiCD38-PD-1loCD27+Tim-3loCD45RO+HLA-DR-
		25	CD57loCD28hiCD38-PD-1+CD27+Tim-3loCD45RO+HLA-DR-
