# Supplementary material for: Multidimensional Clusters of CD4+ T Cell Dysfunction Are Primarily Associated with the CD4/CD8 Ratio in Chronic HIV Infection
Source: PLoS One. 2015 Sep 24;10(9):e0137635. doi: 10.1371/journal.pone.0137635 (PMC4581870; doi:10.1371/journal.pone.0137635)
Supplement: S2 Table — (PDF) [file pone.0137635.s002.pdf]

S2 Table. Clinical parameters correlating with the populations.

	aFLOCK	sFLOCK	Manual
CD4 level	10, 11, 15, 21	4, 12	CD38+HLA-DR+, HLA-DR+, PD-1+
CD4%	10, 11, 13, 15, 21	4, 12	CD38+HLA-DR+, HLA-DR+, PD-1+
CD8 level	15	12	CD38+HLA-DR+, HLA-DR+
CD8%	10, 11, 13, 15	10, 12	CD38+HLA-DR+, HLA-DR+
VL	15	NA	CD38+HLA-DR+
CD4/CD8 ratio	10, 11, 13, 15, 21	4, 10, 12	CD38+HLA-DR+, HLA-DR+, PD-1+
